# Application of Optical Fluorescence Spectroscopy for Studying Bee Abundance in *Tropaeolum majus* L. (Tropaeolaceae)

**DOI:** 10.3390/biology11060887

**Published:** 2022-06-08

**Authors:** Claudemir Antonio Garcia Fioratti, Evaristo Alexandre Falcão, Rosicleia Matias da Silva, Maria do Carmo Vieira, Anderson Rodrigues Lima Caires, Rosilda Mara Mussury

**Affiliations:** 1 Laboratory of Insect-Plant Interaction, Graduate Program in Entomology and Biodiversity Conservation, College of Biological and Environmental Sciences, Federal University of Grande Dourados, Dourados-Itahum Highway, 12th km, Dourados 79804-970, MS, Brazil; claufioratti.ento@gmail.com (C.A.G.F.); rosi-matias09@hotmail.com (R.M.d.S.); 2 Applied Optics Group, College of Exact Sciences and Technology, Federal University of Grande Dourados, Dourados-Itahum Highway, 12th km, Dourados 79804-970, MS, Brazil; evaristofalcao@ufgd.edu.br; 3 Laboratory of Medicinal Plants, College of Agricultural Sciences, Federal University of Grande Dourados, Dourados-Itahum Highway, 12th km, Dourados 79804-970, MS, Brazil; mariavieira@ufgd.edu.br; 4 Optics and Photonics Group, Institute of Physics, Federal University of Mato Grosso do Sul, Campo Grande 79070-900, MS, Brazil; anderson.caires@ufms.br

**Keywords:** capuchinha, floral visitor, visual perception, floral attribute, hymenoptera

## Abstract

**Simple Summary:**

*Tropaeolum majus* L. is a medicinal plant popularly known as *capuchinha*. It produces flowers of different colors and hence attracts various groups of floral visitors. Since these groups may be potential pollinators, they are major for biodiversity conservation. In this regard, our study aimed to verify potential relationships between corolla colors and visiting bee abundance using optical fluorescence spectroscopy and climatic analyses. Orange and yellow flowers were the most visited and showed higher temperatures and fluorescence emissions than did flowers of other colors. This might be due to the presence of compounds such as hydroxycinnamic acid and kaempferol that emit fluorescence around 470 to 620 nm, which are within the visible spectrum for these bee species.

**Abstract:**

*Tropaeolum majus* L. species produce flowers with all sorts of colors, from yellow to red. This work aimed to apply optical fluorescence spectroscopy to study bee abundance in *T. majus*, answering the following questions: (1) do corolla temperature and weather conditions affect the abundance of visiting bee species? (2) do flower color and corolla fluorescence affect the abundance of visiting bee species? (3) do red flowers attract more visiting bees? (4) is there a relationship between bee visits and flower compounds? The bee species *Apis mellifera*, *Paratrigona lineata*, and *Trigona spinipes* were the most observed in *T. majus* flowers. The latter was more active in the morning and preferred orange and yellow flowers. These colors also had higher temperatures and fluorescence emissions than did the red ones and those with yellow-red and orange-red nectar guides. Orange flowers emitted a broadband UV spectrum (between 475 and 800 nm). This range might be due to compounds such as hydroxycinnamic acid, flavonols, isoflavonoids, flavones, phenolic acid, and chlorophyll. Extracts from different *T. majus* corolla colors showed that flowers emit specific fluorescent signals, mainly related to bee color vision and learning, thus acting as a means of communication between bees and flowers. In this way, this information evidences the interaction between bees and *T. majus* flowers, allowing conservation actions for pollinators.

## 1. Introduction

Flower attributes are diverse, e.g., color, odor, shape, temperature, ultraviolet patterns, and nectar availability [1,2,3,4]. Depending on the color, light emitted from petals is one of the main attractants for floral visitors, and therefore pollinators [5]. Nectar guides can act as an optical sign to direct bees to available floral resources [6,7].

The final choice of floral visitors is driven by flower stimuli but depends only on color to thus perform foraging tasks [8,9,10].

Fluorescent signals emitted by flowers have specific functions and can be considered as biocommunication services between insects and plants [11,12,13]; that is, unlike humans, many floral visitors are sensitive to ultraviolet (UV) light emission, rather than the visible band of the spectrum [14,15,16].

The color vision of some insects and spectral properties of flowers has evolved into a mutualistic interaction between plants and their floral visitors, with the color vision system of bees, especially *Apis mellifera* (Linnaeus, 1758) (Hymenoptera: Apidae), being the most understood so far [17]. Since bees differentiate colors, they can better distinguish wavelengths from 400 to 500 nm, wherein the spectral sensitivity of UV, blue, and green photoreceptors overlap [18]. This behavior has also been reported in about 43 taxa from different hymenopterans, which demonstrated a trichromatic vision system with peak sensitivity at UV light (340 nm), blue light (430 nm), and green light (535 nm) [14,19].

Colors considered attractive to most bee species are those perceived by humans as bluish-violet, white, yellow, and orange. This is because the color vision spectrum of bees is within the spectral range of 340 nm to 535 nm; however, longer wavelengths such as red and infrared are barely perceived by bees, i.e., they are considered blind at wavelengths above 700 nm [15,16,19,20,21].

The bee vision system analyzes colors by different photoreceptors, wherein sensitivity to ultraviolet (UV) rays allows detecting and differentiating flowers emitting within this spectrum. To indicate the direction of nectar, some flowers emit fluorescent signals and/or UV radiation through nectar guides, which act as a pathway to floral resources such as nectar and pollen [15,16].

Studies on the attractiveness of fluorescence emitted by flowers to floral visitors may have originated from observations of flowers exposed to ultraviolet radiation [22]. As the association between flower color and floral visitor abundance can be explained by flower fluorescence and affected by weather conditions, our analyses aim to explain the relationship between flower fluorescence and bee visitation through the following questions: (1) do corolla temperature and weather conditions affect the abundance of visiting bee species? (2) do flower color and corolla fluorescence affect the abundance of visiting bee species (3) do red flowers attract more visiting bees? (4) is there a relationship between bee visits and flower compounds?

Given the above, this study aimed to verify whether there is a relationship between the different *Tropaeolum majus* L. (Tropaeolaceae) corolla colors and visiting bee abundance, using optical fluorescence spectroscopy and climatic analysis.

## 2. Materials and Methods

The experiment was carried out at the Garden of Medicinal Plants of the College of Agricultural Sciences, Federal University of Grande Dourados, Dourados, Mato Grosso do Sul, Brazil (54°56′08.5″ W and 22°11′43.7″ S) from August to October 2019. The local climate is classified as tropical monsoon climate *(Am)* [23].

**Figure 1:**
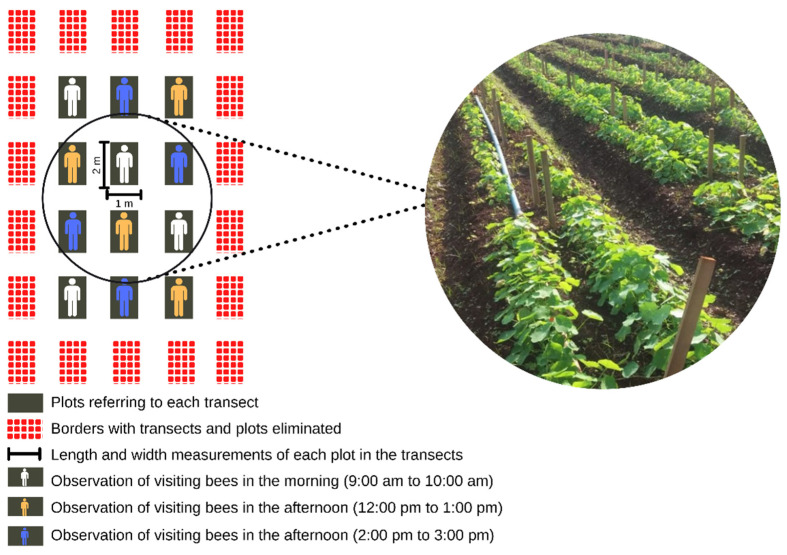
Experimental design of the sampling site containing the transects and plots with plants and flowers of *T. majus* and the hours of observation of the visiting bees.

Seeds of Nasturtium cv. hybrid Alta Dobrada Sortida (Isla^®^) that was grown comes from the seed bank from the Garden of Medicinal Plants of the College of Agricultural Sciences, Federal University of Grande Dourados, Dourados, Mato Grosso do Sul, Brazil. A specimen of the studied species is deposited in the DDMS Herbarium—Dourados—MS, under number 5474. Cultivation was carried out along five 20-m long and 1-m wide transects. During bee behavior evaluations, borders were eliminated, leaving 3 transects of 4 numbered plots (2 m long and 1 m wide) (Figure 1).

### 2.1. Experimental Procedures

**Figure 2:**
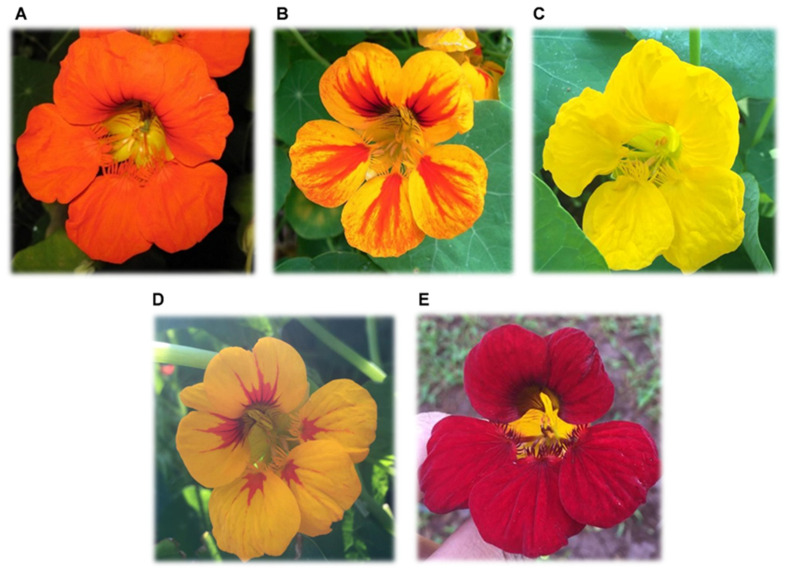
Corollas of *Tropaeolum majus* different colors found in transects and plots during the visit of bees. (**A**) Orange; (**B**) orange-red; (**C**) yellow; (**D**) yellow-red; (**E**) red.

Along the transects, flowers of different corolla colors were monitored, namely: orange, orange-red, yellow, yellow-red, and red (Figure 2).

Visiting bee foraging behavior was evaluated during visits to *T. majus* flowers. Evaluations were carried out for seven weeks, between August and October 2019. To this end, observations were made once a week for 3 h a day, one hour in the morning (9:00 a.m. to 10:00 a.m.) and two in the afternoon (12:00 p.m. to 1:00 p.m. and 2:00 p.m. to 3:00 p.m.), totaling 21 h, thus corresponding to one hour per period. Of the 12 experimental plots, four were drawn each week for a 15-min observation of bee behavior (Figure 1).

Aside from floral visitor behavior, we also evaluated corolla color and flower temperature, measured with a portable infrared thermometer (ScanTemp—Incoterm). Climatic conditions during the experiment (e.g., temperature, wind speed, and relative air humidity) were obtained from an agrometeorological station belonging to the Federal University of Grande Dourados, which is located 7 km apart from the experimental site.

The abundance of floral visitors per corolla color and visiting hours were determined by counting the number of bees, as well as sites and flower colors visited.

### 2.2. Pigment Extraction

Pigments were extracted from 30 *T. majus* flowers of different colors, which were collected, cut, and placed separately into Erlenmeyer flasks containing methanol p.a. at a 1:10 (*m*:*v*) ratio. The flasks were properly sealed with parafilm, wrapped with aluminum foil, and stored in a refrigerator for about three days. Afterward, flower extracts were obtained and diluted in methanol at a 1:1 (*v*:*v*) ratio for fluorescence measurements. The data obtained were compared with the literature findings to determine the compounds present in the flowers and their respective relationship with bee visits.

### 2.3. Fluorescence Measurements

The fluorescence of flower extracts was measured under two different experimental setups. The first consisted of obtaining better fluorescence spectra resolutions and understanding plant mechanisms involved therein. The second aimed to simulate the exposure of *T. majus* flowers to sunlight.

In the first setup, measurements were made using a portable spectrofluorometer. This equipment consists of a 405-nm diode laser, a monochromator (USB 200 FL, Ocean Optics, Dunedin, FL, USA), a Y-type fiber, and a laptop. To this end, samples were placed into a cuvette and positioned about 5 cm from excitation and detection sources. The distance was maintained for all samples. The data collected were used to draw curves representing fluorescence emission peaks divided into three regions. The first region corresponds to fluorescence emission within the range of 450 to 500 nm, the second between 500 and 635 nm, and the third between 635 and 800 nm. This experimental setup provides a better fluorescence signal because it uses a laser as a pump beam.

In the second setup, the excitation–emission matrix spectra of flower extracts were measured by using a Varian Cary Eclipse spectrophotometer, which uses a pulsed xenon lamp (80 Hz; 2-μs pulse width at half peak height) for luminescence excitation and operates at 75 KW peak power. Samples were placed in a 10 mm optical path quartz cuvette, with all four faces polished. The samples were excited with wavelengths between 195 and 550 nm, with Δλ adjusted to 5-nm steps. Emission spectra were then recorded from 450 to 750 nm in 5-nm emission intervals.

### 2.4. Statistical Analysis

The experiment was carried out in a completely randomized resign (CRD) and 3 × 5 × 3 factorial scheme (abundance of visiting bee species × corolla colors × observation times). The Shapiro–Wilk (W) test was used to test data normality, and when it is of non-normality, data were transformed into $\sqrt{x+0.5}$. Results were subjected to analysis of variance (ANOVA), and means were compared by Tukey’s HSD test at 5% probability using Assistat software (v. 7.6).

Visiting bee abundances were correlated with daytime weather conditions and mean floral temperatures. For this, Spearman’s correlation coefficient was used with the aid of the Past software version 3.26. Spearman’s coefficient correlates two quantitative variables, and values between 0.0 and 0.3 (or 0.0 and −0.3) are negligible correlations, between 0.31 and 0.5 (or −0.31 and −0.5) weak, between 0.51 and 0.7 (or −0.51 and −0.7) moderate, between 0.71 and 0.9 (or −0.71 and 0.9) strong, and >0.9 (or <−0.9) very strong. Only correlations with a statistical significance of *p* ≤ 0.05 were considered.

For the preparation of scatter plots between visiting bee species and the temperature of the corollas of *T. majus*, data on the abundance of visiting bee species and the temperature averages of the different colors of the corolla of *T. majus* were used. For the scatter plots between visiting bee species and day temperature, data on the abundance of visiting bee species and day temperatures at three different times were used (9:00 a.m. to 10:00 a.m.; 12:00 p.m. to 13:00 p.m.; 14:00 p.m. to 15:00 p.m.) during the flowering weeks of *T. majus*, in order to relate the dispersion data with the data corresponding to Spearman’s coefficient correlations.

## 3. Results and Discussion

There was no significant triple interaction between the studied factors (abundance of visiting bee species versus corolla color versus observation time) (F = 0.81; DF = 16; *p* > 0.05); however, there was a significant interaction between abundance of visiting bee species and corolla color (F = 9.09; DF = 8; *p* < 0.01) and between abundance of visiting bee species and observation time (F = 3.13; DF = 4; *p* < 0.05). A significant effect was also observed for the isolated factors, abundance of visiting bee species (F = 107.52; DF = 2; *p* < 0.01) and corolla color (F = 46.93; DF = 4; *p* < 0.01).

In total, we counted 1035 bees of the species *Trigona spinipes* (Fabricius, 1793) (Hymenoptera: Apidae), *Apis mellifera* (Linnaeus, 1758) (Hymenoptera: Apidae), and *Paratrigona lineata* (Lepeletier, 1836) (Hymenoptera: Apidae).

The abundance of the three visiting bee species of flowers varied significantly (F = 107.52; DF = 2; *p* < 0.01), and *T. spinipes* was the most abundant species along the transects (7.45 ± 0.88 a), followed by *A. mellifera* (1.89 ± 0.36 b), and *P. lineata* (0.42 ± 0.13 c). Orange (9.00 ± 1.33 a) and yellow (3.90 ± 0.71 b) flowers received more bee visits than did orange-red, yellow-red, and red flowers (1.03 ± 0.30 c; 1.16 ± 0.29 c; 1.33 ± 0.31 c, respectively) (F = 46.93; DF = 4; *p* = < 0.01).

*T. spinipes* bees were abundant in all observed flowers, visiting orange and yellow flowers. *A. mellifera* visited orange flowers, and *P. lineata* visited orange, yellow and red flowers. Finally, *P. lineata* was less abundant (Table 1).

We observed that *T. spinipes* and *A. mellifera* preferred to visit orange and yellow flowers. Results for *Byrsonima variabilis* (Malpighiaceae) were similar to ours, in which large and small bees showed greater preference for orange and yellow flowers during the collection of floral resources (pollen and nectar) [24].

*T. spinipes* preference for different day times for visitation showed its highest abundance, but it did not differ significantly from *P. lineata*. In turn, *A. mellifera* preferred morning hours (9:00 a.m. to 10:00 a.m.). When comparing the day times and visiting bee species, *T. spinipes* differed significantly from the others, preferring the three times for visitation (Table 2).

Regarding visiting hours, *A. mellifera* was more active in the morning (9:00 to 10:00 a.m.), coinciding with the period of higher sugar concentrations in floral nectar [25]. Visitation periods of *A. mellifera* have already been reported in the literature, and pollen and nectar are preferably collected from 8:00 h to 10:00 h and 9:30 h to 10:30 h in the morning, respectively [26,27]. In most plant species, higher pollen concentrations and production are observed in the morning, while nectar is found in the morning and afternoon [28,29,30,31].

The highest visitation frequency in the morning is related to the greater availability of attractive substances in floral structures, given that pollinator visitation has a positive relationship with nectar production [27,32,33]. Although resource availability decreases throughout the day, bees continue to forage flowers but less intensely [34].

**Figure 3:**
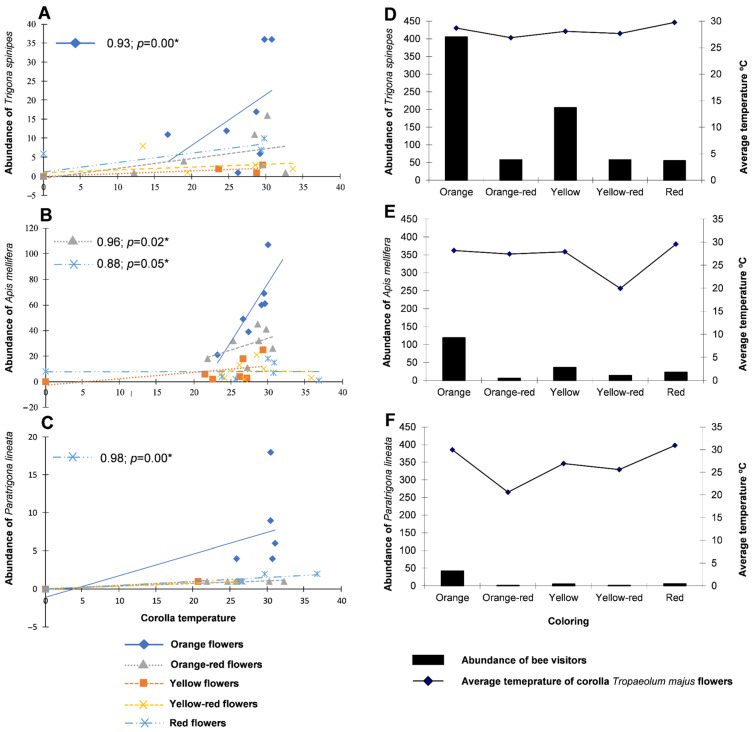
Dispersion corresponding to Spearman’s coefficient correlations between the temperature of the *Tropaeolum majus* corolla and the abundance of *Trigona spinipes* (**A**), *Apis mellifera* (**B**), *Paratrigona lineata* (**C**), and the abundance of *Trigona spinipes* (**D**), *Apis mellifera* (**E**), and *Paratrigona lineata* (**F**) and the average temperature of the corollas of different colors of *Tropaeolum majus* flowers. * Statistical significance of spearman correlation, *p*-value ≤ 0.05.

The correlation between the abundance of visiting bee species and mean corolla temperature during flowering weeks was positive and significant between *T. spinipes* and orange flowers (0.93; *p* = 0.00) (Figure 3A), between *A. mellifera* and orange-red and red flowers (0.96; *p* = 0.02; 0.88; *p* = 0.05) (Figure 3B), and between *P. lineata* and red flowers (0.98; *p* = 0.00) (Figure 3C), with bee abundance increasing according to flower temperature (Figure 3D–F); however, *A. mellifera* had a positive correlation only with low-temperature flowers, while *T. spinipes* and *P. lineata* had positive correlations with high-temperature flowers, mainly *T. spinipes*, which showed greater abundance in *T. majus* transects.

**Figure 4:**
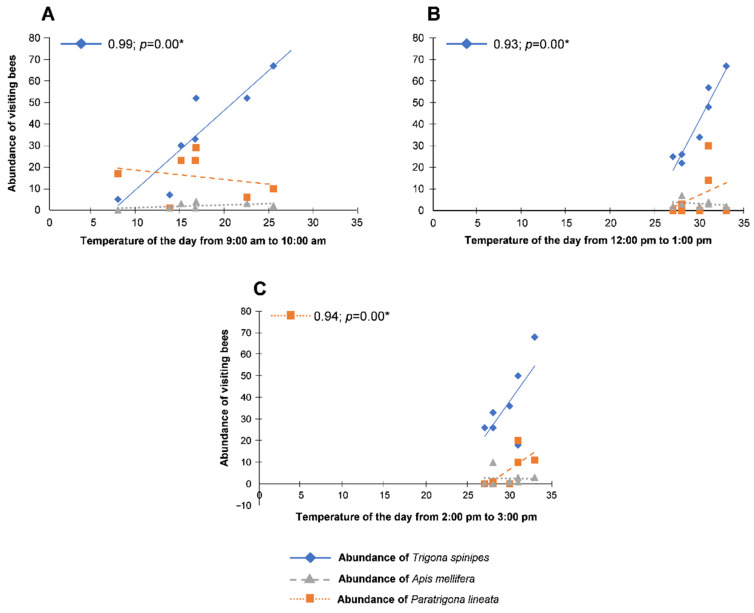
Dispersion corresponding to Spearman’s coefficient correlations between the abundance of visiting bee species and the temperature of the day in the three observation times, from 9:00 a.m. to 10:00 a.m. (**A**), 12:00 p.m. to 1:00 p.m. (**B**), and from 2:00 p.m. to 3:00 p.m. (**C**) during the seven weeks of flowering of *Tropaeolum majus*. * Statistical significance of spearman correlation, *p*-value ≤ 0.05.

Bees can detect and select high-temperature flowers and thus benefit from floral rewards available [35,36]; however, when it comes to the bee visual system, flower colors play a key role in temperature changes [37]. Flowers with more intense colors absorb more heat and hence have higher temperatures; therefore, bees can learn to associate a certain flower color with its temperature, and thus color and temperature increase the efficiency of visitors in acquiring floral resources [36,38]. In comparing visiting bee abundance with weather conditions (temperature, relative humidity, and wind speed) at different times of the day during flowering, we observed a positive and significant correlation only between *T. spinipes* and temperature from 9:00 a.m. to 10:00 a.m. (0.99; *p* = 0.00) (Figure 4A); for the other species of bees, no correlation was observed for relative humidity and wind speed. On the other hand, between 12:00 p.m. and 1:00 p.m., only *T. spinipes* showed a positive and significant correlation with temperature (0.93; *p* = 0.00) (Figure 4B), relative humidity (−0.77; *p* = 0.04), and wind speed (0.80; *p* = 0.03). For the other bee species, no correlation was observed. Between 2:00 p.m. and 3:00 p.m., only *P. lineata* showed a positive and significant correlation with the climatic condition (temperature) (0.94; *p* = 0.00) (Figure 4C); for the other bee species, no correlation was observed.

Given the above, we can confirm that visiting bee vision mainly uses UV light to identify flowers, sun orientation, and control flight positioning [39,40,41]. Furthermore, bees can adapt their vision to changes in UV emission ratio within the light spectrum [42].

Another factor influencing petal colors is secondary metabolites [43,44]. The highest flavonoid and anthocyanin contents were found in *T. majus* flowers of red color, while orange and yellow flowers had the highest carotenoid contents [45,46].

**Figure 5:**
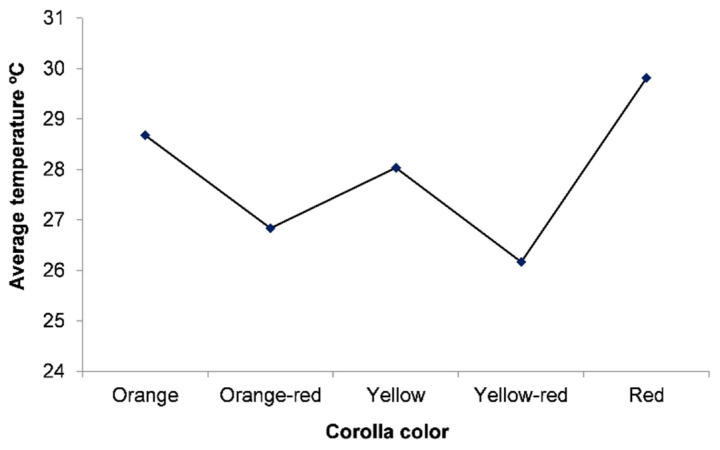
Corolla temperature averages of different *Tropaeolum majus* flower colors.

The contents of bioactive compounds in flowering plants, such as carotenoids, can be influenced by several abiotic factors. Of these, temperature and luminosity are paramount [47] in changes in visitation abundance, as high luminosity and high temperatures favor bee visitations [27]. At low temperatures, high-wind and low-light conditions reduce the ability of bees to fly [48,49]. These results corroborate findings in the field (Figure 3D–F), in which orange, yellow, and red *T. majus* flowers had higher temperatures and the highest bee abundances, especially the orange ones. Conversely, orange-red, and yellow-red flowers had lower temperatures and received fewer visitations (Figure 5).

**Figure 6:**
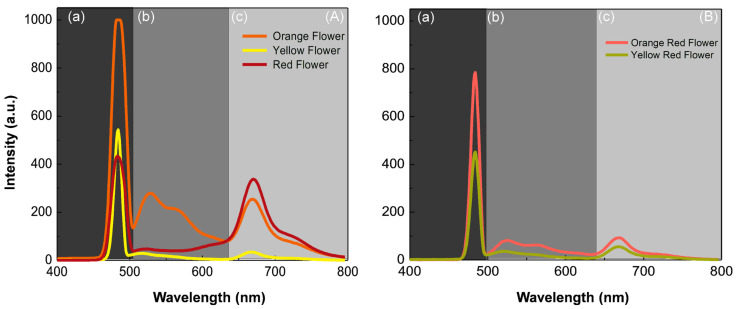
Fluorescence intensity (in arbitrary units, a.u.) at different wavelengths of orange, yellow and red corolla flowers (**A**), nectar-guided flowers (orange-red and yellow-red) (**B**) of *Tropaeolum majus.* Fluorescence intensity corresponding to fluorophores and flavonols (**A**(**a**),**B**(**a**)—black); β-carotene and anthocyanin (**A**(**b**),**B**(**b**)—dark gray); and chlorophyll (**A**(**c**),**B**(**c**)—light gray).

The fluorescence test of *T. majus* flowers showed three intensity bands of different wavelengths (Figure 6), namely: first band (between 450 and 500 nm; Figure 6A(a),B(a)), with a peak centered around 480 nm due to the presence of fluorophores (e.g., hydroxycinnamic acid) [50] and flavonols (e.g., myricetin, quercetin, and kaempferol) [51]; second band (between 500 and 635 nm; Figure 6A(b),B(b)), with two peaks, one at about 528 nm and another at about 564 nm, which may be related to the presence of β-carotene, with greater intensity compared to that of anthocyanin, respectively [52,53]; third band (Figure 6A(c),B(c)), with two peaks, one at 670 and another at 722 nm, attributed to chlorophyll *a* and *b* fluorescence, respectively [54]. Thus, we noted that red corolla flowers have a peak at 480 nm, which is smaller than those of orange and yellow corolla flowers (Figure 6A(a)), and a larger peak at 670 nm, which is attributed to chlorophyll *a* and *b* fluorescence (Figure 6A(c)).

Orange corolla flowers showed a broad fluorescence band from 475 to 800 nm (Figure 6A). We found that, between 400 and 600 nm, fluorescence was due to the presence of compounds such as hydroxycinnamic acid, flavonols, isoflavonoids, flavones, and phenolic acid, while between 600 and 800 nm, it was due to chlorophyll [54]. As most of these compounds are present in *T. majus* flowers, the fluorescence spectrum pattern (Figure 6) agrees with their contents [50,51]. Orange flowers of *T. majus* have the largest amounts of kaempferol, followed by red and yellow ones [51]. Among the flavonols in paprika samples, kaempferol had the highest fluorescence intensity at excitation, around 405 nm, whereas quercetin and myricetin had much lower intensities [55]; therefore, the fluorescence of the region (a) in Figure 6 is determined by kaempferol in orange and red flower extract samples, greater in orange than in red flowers. The spectrum in this region of yellow flowers, which had the least amount of these flavonols, was determined by their largest amount of hydroxycinnamic acid.

Since all fluorescence measurements were made under similar experimental conditions, such as concentrations and sample-detector distance, one can see in Figure 6A,B that orange *T. majus* flowers had the highest intensity for the three analyzed bands.

**Figure 7:**
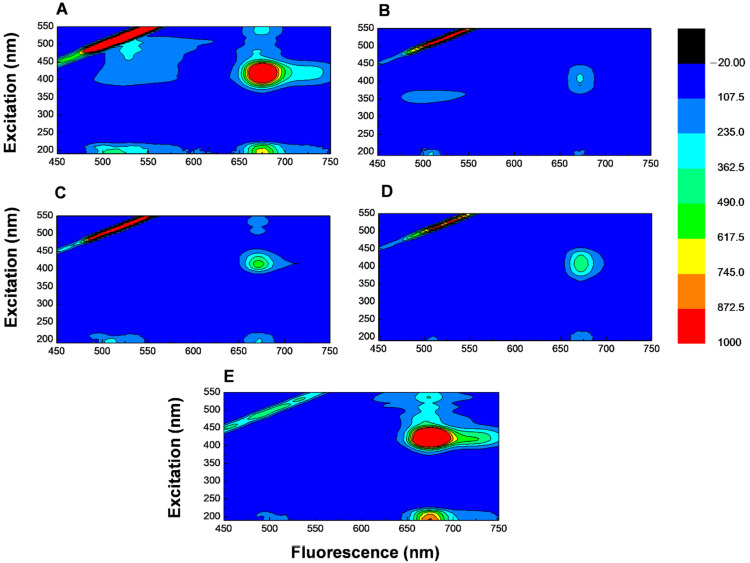
Excitation–emission matrix spectra of *Tropaeolum majus* flower extracts in orange (**A**), yellow (**B**), orange-red (**C**), yellow-red (**D**), and red (**E**) colors.

To represent *T. majus* flower extract spectra varying excitation and emission, the experimental results were presented up to 650 nm, aiming to encompass only the eyesight of bees (Figure 7). This is because many insects, especially bees, have a trichromatic vision system [56]; that is, they prefer colors with fluorescence emission between 320 and 650 nm [15,16,21,56,57].

Among the flowers observed, the orange and yellow ones received a greater abundance of insects, and hence relevant fluorescence emissions from 470 to 620 nm (Figure 7A,B), which is due to carotenoids such as β-carotene [52,53] that absorb UV light in the blue band (430 nm) and emit yellow (580 nm), orange (600 nm), and red (700 nm) wavelengths [12,58]. Once hymenopterans are trichromatic, yellow-colored surfaces are more visible and contrast with vegetation green background [56], thus highlighting bee preference for orange and yellow *T. majus* flowers for visitation.

The least preferred flowers by bees were the orange-red and yellow-red ones (Figure 7C,D), showing low fluorescence emissions for carotenoids (around 480 to 540 nm) and high emissions for chlorophyll (around 650 and 720 nm). Red flowers (Figure 7E) showed low fluorescence emission between 480 and 520 nm and higher emissions between 620 and 750 nm, with the band between 600 and 800 nm being due to chlorophyll [54], which absorb UV light in the blue (430 nm) and red (700 nm) bands and emit green wavelengths (535 nm) [59,60].

Bees do not have a red-light receptor in the 700 nm band because their vision is restricted to short wavelengths between UV (300 nm) and orange (690 nm), while red and infrared (IR) are in a spectral range above 700 nm [36], explaining the low bee visits in these flowers. Thus, the green fluorescence color emitted by red *T. majus* flowers resembles green foliage dispersed in the environment, as leaves also present a chlorophyll compound with green fluorescence emission [19,24,56]; therefore, red corolla flowers were not in evidence, explaining the low bee visit abundances in orange-red, yellow-red, and especially red flowers, which had higher emissions. It is noteworthy that the odor exhaled by *T. majus* stamens attracts pollinators, serving as a cue to find these unattractive flowers [25].

Our results support the hypothesis that flower fluorescence measurements indicate UV wavelength emissions, which stimulate insect eye photoreceptors [61]. They also prove that bees visit fewer red flowers due to their higher emissions (above 700 nm) and high chlorophyll fluorescence index in this band. Furthermore, our outcomes suggest that flower fluorescence pattern is an important signal in flower recognition by visiting bees [15,16,24,61]; therefore, our findings raise new possibilities in the perception of floral visitors since fluorescence was not considered important in attracting bees to different *T. majus* flowers.

As a future perspective, further studies are needed to measure the fluorescence of nectar and pollen, as well as their relationship with floral reward availability and attraction of visiting bees to different *T. majus* flower colors.

## 4. Conclusions

Corolla fluorescence affects the abundance of visiting bee species, with orange and yellow flowers being the most visited by bees due to their higher temperature and fluorescence emission compared to red, yellow-red, and orange-red flowers. Climatic conditions also affect visitation by bees but depend on daytime and bee species. There is a relationship between visits by bees and floral compounds since orange-red flowers have kaempferol and yellow ones have less of these flavonols, and their spectrum is determined by a greater amount of hydroxycinnamic acid. Although visited, the red flowers must appear black to the bees, as the light emitted by them is beyond the spectral range of the bees, above 700 nm.

## Figures and Tables

**Table 1:** Abundance of visiting bee species in *Tropaeolum majus* flowers of different colors.

	Orange	Orange-Red	Yellow	Yellow-Red	Red
*Trigona spinipes*	19.33 ± 2.46 aA *n* = 406	2.76 ± 0.78 aC *n* = 58	9.76 ± 1.31 aB *n* = 205	2.76 ± 0.65 aC *n* = 58	2.62 ± 0.70 aC *n* = 55
*Apis mellifera*	5.67 ± 1.33 bA *n* = 119	0.29 ± 0.12 bB *n* = 6	1.71 ± 0.52 bB *n* = 36	0.67 ± 0.40 bB *n* = 14	1.10 ± 0.48 abB *n* = 23
*Paratrigona lineata*	2.00 ± 0.54 cA *n* = 42	0.05 ± 0.05 bB *n* = 1	0.24 ± 0.10 bAB *n* = 5	0.05 ± 0.05 bB *n* = 1	0.29 ± 0.12 bAB *n* = 6
CV(%)	50.57				

Means followed by the same letter do not differ statistically from each other, lowercase in the columns and uppercase in the rows, when compared by Tukey’s test at 5% probability; *n* = number of individuals; CV = coefficients of variation.

**Table 2:** Abundance of visiting bee species in *Tropaeolum majus* flowers at different day times.

	9:00 a.m.–10:00 a.m.	12:00 p.m.–1:00 p.m.	2:00 p.m.–3:00 p.m.
*Trigona spinipes*	7.03 ± 1.89 aA *n* = 246	7.97 ± 1.35 aA *n* = 279	7.34 ± 1.28 aA *n* = 257
*Apis mellifera*	3.11 ± 0.76 bA *n* = 109	1.34 ± 0.57 bB *n* = 47	1.20 ± 0.48 bB *n* = 42
*Paratrigona lineata*	0.40 ± 0.11 cA *n* = 14	0.66 ± 0.25 bA *n* = 23	0.51 ± 0.30 bA *n* = 18
CV (%)	50.57		

Means followed by the same letter do not differ statistically from each other, lowercase in the columns and uppercase in the rows, when compared by Tukey’s test at 5% probability; *n* = number of individuals; CV = coefficients of variation.

## Data Availability

The data presented in this study are available on request from the corresponding author.
